# “Back to the future”: Influence of beliefs regarding the future on TTO answers

**DOI:** 10.1186/s12955-015-0402-6

**Published:** 2016-01-12

**Authors:** F. E. van Nooten, N.J.A. van Exel, D. Eriksson, W.B.F. Brouwer

**Affiliations:** Institute of Health Policy & Management, Erasmus University Rotterdam, Rotterdam, The Netherlands; Institute for Medical Technology Assessment, Erasmus University Rotterdam, Rotterdam, The Netherlands; Quantify Research, Stockholm, Sweden

## Abstract

**Background:**

A common approach to obtain health state valuations is the time-tradeoff (TTO) method. Much remains unknown regarding the influence of responder characteristics on TTO answers. The objective of this study is to increase understanding of the influence that beliefs regarding future health and death, as well as desires to witness certain life events, have on respondents’ health state valuations.

**Methods:**

An online survey was designed, including three TTO questions using a 10 year timeframe. Moreover, respondents completed demographic questions, the Health-Risk Attitude Scale (HRAS), the Expectations Regarding Aging (ERA) questionnaire, questions about beliefs regarding future health (i.e. life expectancy) and death (i.e. fear of death, belief in life after death and opinion about euthanasia), and about important life events taking place within the TTO timeframe. Regression analyses were performed in order to assess the influence of these different variables.

**Results:**

One thousand sixty-seven respondents were included in the analyses. The following variables were significantly associated with years traded off: ERA mental health (decrease), ERA physical health (increase), HRAS (increase), support for euthanasia (increase), fear of death (decrease) and consideration of an important life event (decrease). The explained variance of the final model was low (0.08).

**Conclusion:**

TTO responses may be influenced by considerations of future health, including life events and attitudes regarding health risks and death. Further investigation of TTO responses remains warranted.

**Electronic supplementary material:**

The online version of this article (doi:10.1186/s12955-015-0402-6) contains supplementary material, which is available to authorized users.

## Background

Several countries use cost-effectiveness analyses in the context of deciding on the reimbursement and funding of new medical technologies (e.g. United Kingdom, Sweden, the Netherlands). For the effectiveness component, some authorities prefer the use of quality adjusted life years (QALYs), as these are believed to allow for a universal comparison across diseases areas [1]. QALYs combine length and quality of life, with the latter normally expressed as a value between 0 (dead) and 1 (perfect health). Societal preferences obtained in the general public typically underlie these values [2, 3].

Different valuation techniques are used to obtain health state valuations from the general public. The most widely used approach is the time-trade off (TTO) method. In a TTO exercise, a respondent is presented with two health streams. One of the health streams is a fixed lifespan (e.g. 10 years) lived in some imperfect health state ‘A’. The other stream entails a shorter lifespan but lived in perfect health. Respondents are subsequently asked to indicate the minimum number of years lived in perfect health required to become indifferent between the two streams. If a respondent is not willing to live shorter in perfect health relative to the imperfect health state A, its value is assumed to be equal to that of perfect health (with value 1). When a respondent is willing to give up all remaining years, the value of health state A is considered to be equal to being dead (with value 0) [4]. If a respondent indicates to consider 6 years in perfect health (with value 1) equal to 10 years in state A, the value of health state A is assumed to be equal to 0.6. In this way, states of health impairment can be assigned a value between 0 and 1, with a higher score indicating a better health state. Other variants of the TTO exist for health states worse than death. In those cases the lead time TTO should be employed (Devlin et. al. 2011, Attema et. al. 2013)

Despite the widespread use of TTO as valuation method for health state utilities in economic evaluations, relatively little is known about which characteristics of respondents are associated with responses to time trade-off exercises. Most of the previous research was focused on the typical demographics like age, gender, marital status and education [5–7], but findings have been mixed both in terms of the direction of influences and their statistical significance. For example, several authors studied the relationship between age and TTO values as part of their analyses. Augestad et al. [8], Best et al. [9] and Hsu et al. [10] found a positive relationship, while Ayalon and King-Kallimanis [11], Shimizu et al. [12], and Zarate et al. [13] found negative relationships. Similar findings were found for gender. Brown et al. [14], Gupta et al. [15] and Tamayama et al. [16] found positive relationships with TTO scores and female gender, whereas Wells et al. [17] and Rutten-van Mölken et al. [18] found the opposite. These mixed findings may relate to numerous aspects, amongst others differences in studied populations (e.g. patient or general samples, cultural differences) and in methodology. Another explanation may be that unobserved variables, other than the standard demographic characteristics, influence TTO scores and confound some of the observed relationships.

Loss of future life years is a critical element of a TTO exercise, as length of life is traded off against quality of life. Therefore, attitudes towards future health and death for instance could play an important role. Some research has been performed in this direction by investigating the influence of subjective remaining life expectancy (SLE; calculated as the age the respondent assumes to reach minus current age). SLE turned out to have a negative relationship with the numbers of years traded-off in a TTO exercise. This means that a higher SLE is associated with a lower the number of years traded-off [19–22]. Next to SLE, beliefs regarding life after death have been suggested to influence TTO values [18].

Given the widespread use of TTO and its potential impact on reimbursement decisions, better understanding of such associations is important, not only to understand what drives TTO answers, but also for purposes of representative sampling. If these associations exist, comparing TTO scores from one study to another might not be valid if the sampled populations differ with respect to these variables.

The aim of this study is to obtain more insight in this underexplored relation of responses to TTO exercises with beliefs regarding future health and death.

## Methods

A questionnaire was administered online by a survey company to a representative sample of the Dutch general public in the range 18 to 65 years, in terms of age, gender and level of education. Respondents who completed the survey in less than 15 min were considered to have devoted too little attention to the questions and, consequently, were excluded from the analyses. (This threshold for speeding through the questionnaire was based on the distribution of completion times in the pilot test.) The methods are described in more detail in Van Nooten et al. [23], who used the same dataset but focused on other parts of the same questionnaire.

The questionnaire first covered common demographic characteristics such as age, gender, marital status, nationality, education, having children, followed by questions regarding current health status, using EQ-5D and EQ-VAS [24]. Next, respondents were asked to rank order six health states. Five of the six health states were described using the EQ-5D descriptive system. The five health states were perfect health, own current health status (as respondents reported previously in the EQ-5D), and three states of health impairment chosen to represent a broad range across health states (see Additional file 1 for an explanation of these health states). The sixth health state was labeled ‘dead’. After rank ordering these health states, respondents were asked to rate them on a visual analog scale (VAS) ranging from 0 (worst imaginable health state) to 100 (best imaginable health state). Finally, respondents were asked to perform three TTO exercises with a 10 year timeframe, for the three imperfect health states. The three states were presented in the order the respondent had ranked them (among own current health, perfect health and dead), from highest to lowest. The flow of the TTO questions is described in detail in Additional file 2.

After the TTO exercises, respondents were asked whether they had thought that the specified period of 10 years would start immediately. If answered affirmatively, respondents were asked if they had thought of a minimum period they wanted to stay alive, for example to witness a certain event or to reach a certain age, regardless of health). In case this question was answered affirmatively, respondents were asked to describe the event.

Respondents also answered a number of questions regarding beliefs about future health. First, respondents reported their subjective life expectancy (SLE) by providing a point estimate of expected lifetime.

Secondly, respondents completed the Health-Risk Attitude Scale (HRAS), which was developed to understand health related risk attitude [25]. The HRAS consists of 13 items, which are scored on a 7 points Likert scale (1 = totally disagree; 7 = totally agree). The 13 items are statements about how much risk respondents are willing to take with their health (for example: “When I look back at my past, I think that, in general, I did take risks with my health.” or, “Safety first, where my health is concerned”). The item scores are summed to obtain a total score ranging from 13 to 91, with a higher score indicating more risk seeking in the health domain [25].

Thirdly, respondents completed the Expectations Regarding Aging (ERA) survey [26]. The ERA consists of three scales (i.e. expectations regarding physical health, expectations regarding mental health, and expectations regarding cognitive function, all related to aging) with four items each, making a total of 12 items, with 4 response options (1–4). The total score for each scale is calculated by summing the responses to each question, which is then rescaled to a range of 0–100, with higher scores indicating higher (that is, better) expectations regarding aging in the physical, mental health and cognitive function domains [26].

Finally, respondents were asked several questions regarding beliefs about death, because the TTO involves shortening life duration in order to improve quality of life. Respondents were asked: “Do you believe in life after death?”, with the following answering possibilities (1) “no, I don’t believe in life after death”, (2) “yes, I believe heaven exists”, (3) “yes, I believe in reincarnation”, and (4) “yes, other (please explain)”. Then respondents were asked: “Are you afraid of death?” using a 0–100 visual analog scale, with 0 representing no fear and 100 extreme fear. Finally, respondents were asked: “What is your attitude regarding euthanasia?” The following four answering possibilities were provided: (1) “I think that euthanasia should not be allowed under any circumstances”, (2) “I think that euthanasia should only be allowed under very strict circumstances (for example in case of unbearable suffering without any hope for the future)”, (3) “I think that euthanasia should be allowed after careful consideration and with professional support”, and (4) “I think that people should be free to opt for euthanasia”.

### Data analysis

Correlations coefficients were computed and were used to understand the relationship between the different variables included in this study, both the demographic variables (i.e. age, gender, partnership status, education, quality of life, number of children) and the beliefs regarding future health and death (i.e. SLE versus event, HRAS, ERA, fear of death, belief in life after death, attitude towards euthanasia).

Next, regression analyses were conducted. The dependent variable in the regression analyses was the number of years a respondent was willing to give up from the 10 year timeframe in order to regain full health, calculated by subtracting the TTO answer from 10. Remaining SLE was calculated by subtracting the actual age of the respondent from expected age of death. The variable for life after death was a dichotomous variable, in which the response options “yes, I believe heaven exists”, “yes, I believe in reincarnation” and “yes, other (please explain)” were classified as 1 and “no, I don’t believe in life after death” as 0. The variable called “event” was created in the following way: if respondents had answered affirmatively to both the question regarding whether they had thought of a minimum period they wanted to stay alive to witness a life event as well as the question whether they thought the 10 year period would start immediately, the variable event was defined as 1, otherwise as 0. Moreover, a dichotomous variable for euthanasia was created in which “I think that euthanasia is not allowed under any circumstances” was coded 1, whereas “I think that euthanasia is only allowed under very strict circumstances (for example in case of unbearable suffering without any hope for the future)”, “I think that euthanasia is allowed after careful consideration and with professional support”, and “I think that people should be free to opt for euthanasia” were coded 0.

### Models

First, we estimated a base model, including the commonly investigated variables in the context of explaining TTO answers: age, gender, partnership status, having children, educational status, own health using the EQ-5D or VAS and SLE [23].

Next, we expanded the base model with the variables related to beliefs regarding future health and death, and any important life events respondents wanted to witness within the 10 year timeframe of the TTO exercise. First, a principal-components factor analysis was conducted to explore the structure in the relationships between these additional variables (ERA mental, ERA cognitive and ERA physical scales, HRAS, belief in life after death, fear of death, views on euthanasia, and the wish to experience a specific future event). The factor analysis allowed for combining correlated variables into independent groups of variables, which were then added separately to the base regression model to determine which individual variables could further explain the TTO scores. Those variables that reached statistical significance (*p* < 0.05) in explaining years traded off in the TTO exercises were included in the final model.

The data were analyzed using random-effects models to account for the repeated TTO measures. Confidence intervals were obtained via bootstrapping, as the data were not normally distributed. All analyses were conducted in Stata/IC version 12.1 for Windows (StataCorp LP, College Station, TX, US).

## Results

### Responder characteristics

From the original 1223 respondents who completed the survey, 156 were excluded for speeding through the questionnaire, which left a total of 1067 respondents for the analyses. Table 1 presents the demographics of this sample. The mean age of the total responder population was 43 years, half were male and the mean VAS score was 75. In responding to the TTO questions, 16 % of respondents had considered an event they wanted to witness or an age they wanted to reach. In 62 % of these cases, the event was related to children or grandchildren (e.g. birth, seeing them grow up to be independent, or attending their wedding). Five percent of the respondents were against euthanasia under all circumstances and 56 % of the respondents believed in life after death. On a VAS scale from 0 to 100, the mean fear of death score was 36. The mean ERA score for the mental health subdomain was 60.4, for the physical health subdomain 31.1 and cognitive function 38.5. The average HRAS score was 44.9.

**Table 1:** Demographics of respondents

	All respondents (*n* = 1067)
Age,years (mean, (SD), range)	43.2 (13.64) 18–65
Gender,male	50.2 %
VAS (mean, (SD))	75.0 (16.59)
High education	30.9 %
Married	49.0 %
Living together	15.3 %
Children (yes)	60.2 %
SLE,years (mean, (SD))	37.8 (17.21)
ERA Mental Health (mean, (SD), range)	65.4 (22.33) 0–100
ERA Physical Health (mean, (SD), range)	31.1 (17.45) 0–100
ERA Cognitive Health (mean, (SD), range)	40.3 (19.91) 0–100
HRAS (mean, (SD), range)	44.9 (9.63) 15–84
Fear of death,scale 0–100 (mean, (SD), range)	35.5 (30.14) 1–100
Support for euthanasia (allowed)	95.1 %
Belief in life after death (yes)	55.8 %
Considered Event (yes)	15.8 %

In general, the correlations between the demographic variables (i.e. age, gender, partnership status, education, quality of life, number of children), beliefs regarding future health and death (i.e. SLE, euthanasia, HRAS, ERA, fear of death, belief in life after death) and staying alive to witness a life event were weak, ranged between −0.2 and 0.2.

### Influence of future expectations

Table 2 first shows the results of the base model. Positive coefficients indicate an increase in the number of years traded, while negative coefficients indicate a decrease in the number of years traded. In this model, age (decrease), being male (increase), living together (increase), having children (decrease), quality of life (VAS) (increase) and subjective life expectancy (decrease) were statistically significantly associated with years traded-off. The association with education level and being married did not reach statistical significance.

**Table 2:** Results including previous used variables (dependent variable: Years traded-off)

	Base Model			Final Model		
R^2^	0.04			0.08		
	Coefficients	Bias corrected 95 % Confidence interval		Coefficients	Bias corrected 95 % Confidence interval	
Age	**−0.040**	**−0.049**	**−0.032**	**−0.041**	**−0.054**	**−0.028**
Male	**0.275**	**0.117**	**0.426**	0.032	−0.184	0.259
VAS	**0.005**	**0.000**	**0.009**	0.006	−0.001	0.013
Highest Education	0.045	−0.12	0.218	0.094	−0.132	0.311
Married	−0.221	−0.422	0.019	−0.169	−0.433	0.093
Living together	**0.369**	**0.140**	**0.595**	**0.327**	**0.030**	**0.635**
Children	**−0.343**	**−0.541**	**−0.149**	**−0.266**	**−0.541**	**−0.007**
SLE	**−0.028**	**−0.035**	**−0.021**	**−0.024**	**−0.034**	**−0.014**
ERA mental health				**−0.009**	**−0.015**	**−0.004**
ERA physical health				**0.007**	**0.000**	**0.013**
HRAS				**0.017**	**0.007**	**0.029**
Fear of death				**−0.009**	**−0.013**	**−0.006**
Support for euthanasia (allowed)				**0.228**	**0.096**	**0.360**
Belief in life after death (yes)				−0.209	−0.417	0.010
Considered event (yes)				**−0.581**	**−0.871**	**−0.284**

Bold are statistically significant based on confidence intervals

The factor analysis resulted in three groups of variables to be added to the base model: 1) expectations regarding aging (the ERA mental, cognitive and physical scales), 2) beliefs regarding death (fear of death, belief in life after death), and 3) health related risk attitude (HRAS), and consideration of a future life event. Support for euthanasia did not load into any of the factors and showed limited variation (only 5 % of the population was against euthanasia). It was added separately to the base case model.

These three groups of variables were first added to the base case model independently in order to assess their separate association with years traded off. For the variables in group 1 (expectations regarding aging), only the ERA mental and physical scales proved to be significantly associated with years traded off. For the variables in the other two groups (group 2: beliefs about death; group 3: health risk attitude) all variables were statistically significantly associated with years traded off when added separately to the base model. When added, support for euthanasia also proved to be significantly associated with years traded off. Therefore, the following variables were included in the final model: ERA mental and physical, fear of death, belief in life after death, HRAS and consideration of a future life event. When these variables were jointly added to the base case model, the following statistically significant results were observed: ERA mental health (increase), ERA physical health (increase), fear of death (decrease), HRAS (increase), consideration of a future life event (increase) and support for euthanasia (increase) (Table 2). Belief in life after death was not statistically significantly associated with years traded off (Table 2). Adding these additional variables to the model resulted in two variables from the base model to lose their significant association with years traded off (i.e. VAS and gender). Furthermore, compared to the base model, the variance explained by the final model doubled, although the absolute value of *R*^2^ remained modest.

## Discussion

While different responder characteristics can influence TTO responses [5–7], previous research has focused mainly on demographics like age, gender and marriage [5–7]. This research has introduced a new category of variables, but has not been able to explain much more of the variance in TTO responses than previous studies. The objective of this study was to investigate the influence that beliefs about future health and death, and desires to witness certain life events, have on TTO responses. Since TTO exercises ask respondents to trade-off (future) quality of life and life duration, beliefs and desires regarding the future may well play a role in final responses. It already has been observed that expectations about length of life play a role in TTO responses [19–23]. Given the importance of adequate health state valuations, it seemed worthwhile to explore this further. We found that both beliefs about future health and death and desires to witness a life event indeed had a significant, though modest, influence on years traded off in a TTO exercise. The effect sizes of these newly identified variables influencing TTO scores are small, however no smaller than previously identified variables (e.g. age and gender). The explained variance increased compared to the base case model showing that the newly identified variables provide more clarification of factors influencing TTO scores. This also indicate that there is probably not one variable influencing TTO scores, but that there are many pieces to this puzzle that need to be put together.

Before discussing the implications of this study, several limitations need to be noted. First, we used a web-based design in our study, which may have had consequences on, for instance, the involvement of respondents in the questionnaire and did not allow face-to-face explanations of questions. Based on a pilot test of the questionnaire we determined a minimum acceptable completion time of 15 min, in order to limit the effect of speeding through the questionnaire and low involvement. Second, there was no separate valuation exercise for health states ranked as being worse than dead, which may have influenced our results. Valuing worse than dead states using a distinct valuation exercise was considered cognitively demanding and alternative methods, which allow better than dead and worse than dead states to be valued in one exercise, such as the lead time TTO [27] may have the same problem and require further validation. Third, only a single iteration was performed in the TTO exercise, instead of a more common repeated choice (‘ping-pong’) exercise, which could have allowed for a more precise estimation of the responders’ indifference point in the trade-off exercise, and to different results [28]. Fourth, this study was performed in the Netherlands, where certain values, norms and beliefs about life and death may be different from other countries. Therefore, extrapolating these results to other countries requires caution. For example, the vast majority of the Dutch society is not against euthanasia (only 5 % of the responders in this study were against euthanasia under all circumstances). This may not be the case in other countries. Performing a similar study in countries where people generally hold different beliefs about life after death, could lead to different results. Fifth, this study only included respondents up to the age of 65 years. The elderly, however, may exert preferences regarding for example euthanasia and beliefs about the future that are different compared to young adults due to age and lived experience. Hence a study including more respondents above the age of 65 could provide different results.

Notwithstanding these limitations, our results showed an influence of beliefs about future health and death on TTO answers. Given the limited knowledge so far in this area, these findings add to the existing literature. First of all, respondents who have higher expectations about future mental health, who aim to stay alive in order to witness a particular life event and those who are afraid of death, all traded off fewer years in the TTO exercises. Respondents who were not opposed to euthanasia were willing to give up more years, as well as those who were more risk seeking in the health domain. Although the explained variance of the final model remained low, the influence of support for euthanasia and the desire to witness a particular life event was remarkably high compared to other statistically significant variables in the model.

Research investigating the influence of beliefs about death on TTO scores is scarce. Rutten-van Molken et al. [18] found that beliefs about life after death were significantly associated with TTO scores. In our study this association was not statistically significant in the final model. This difference may relate to differences in studied populations, included variables and differences in TTO design. Furthermore this study also included fear of death and euthanasia, which although not the same as beliefs about life after death, could have mitigated the effect. More research in this area appears to be warranted.

Another interesting finding was that the ERA variable mental health was negatively associated with years traded off, suggesting that when responders have higher expectations regarding mental aging they are willing to give up fewer years. Although not in line with the instruction and intention of the TTO exercise, which specified a stable health state for the 10 year timeframe and should therefore render own expectations irrelevant, this implies that future years are more easily traded when one expects these will be spent in relatively poor mental health. However, higher expectations regarding physical health were associated with *more* years sacrificed. This rather counterintuitive result may be related to the skewed distribution of ERA physical health (see Table 1).

It should be noted that a 10-year timehorizon was applied for the health state valuation in this study. However it could be expected that in a TTO exercise with a longer time horizon, e.g. life time, the associations highlighted in this study may play an even greater role and this should be investigated.

## Conclusion

This study showed that TTO scores are associated with considerations of future life events and beliefs about future health and death, next to the more common demographic variables observed in the literature like age and SLE. However, the overall impact on providing a better understanding of what drives responders to make choices in TTO scoring remained limited. Therefore, much remains unknown and more research in this area is warranted. Nonetheless, the results of this study suggest that beliefs about the future can be influential in TTO exercises and should therefore be considered in future research on predictors of TTO responses. These findings may also be relevant in sampling for representative societal valuations of health states.

## Additional files

Additional file 1:
**Health states used in the TTO exercise, in addition to ‘own current health status’ and ‘dead’.** (DOCX 27 kb) Additional file 2:
**Flow of TTO question.** (DOCX 31 kb)
